# In-situ observation of plasmon-controlled photocatalytic dehydrogenation of individual palladium nanoparticles

**DOI:** 10.1038/s41467-018-07108-x

**Published:** 2018-11-07

**Authors:** Michal Vadai, Daniel K. Angell, Fariah Hayee, Katherine Sytwu, Jennifer A. Dionne

**Affiliations:** 1 Department of Materials Science and Engineering, Stanford University, Stanford, CA 94305 USA; 2 Department of Electrical Engineering, Stanford University, Stanford, CA 94305 USA; 3 Department of Applied Physics, Stanford University, Stanford, CA 94305 USA

## Abstract

Plasmonic nanoparticle catalysts offer improved light absorption and carrier transport compared to traditional photocatalysts. However, it remains unclear how plasmonic excitation affects multi-step reaction kinetics and promotes site-selectivity. Here, we visualize a plasmon-induced reaction at the sub-nanoparticle level in-situ and in real-time. Using an environmental transmission electron microscope combined with light excitation, we study the photocatalytic dehydrogenation of individual palladium nanocubes coupled to gold nanoparticles with sub-2 nanometer spatial resolution. We find that plasmons increase the rate of distinct reaction steps with unique time constants; enable reaction nucleation at specific sites closest to the electromagnetic hot spots; and appear to open a new reaction pathway that is not observed without illumination. These effects are explained by plasmon-mediated population of excited-state hybridized palladium-hydrogen orbitals. Our results help elucidate the role of plasmons in light-driven photochemical transformations, en-route to design of site-selective and product-specific photocatalysts.

## Abstract (web summary)

While plasmonic nanoparticles offer new opportunities for photocatalysis, understanding the underlying effects remains challenging. Here, the authors visualize plasmon-driven dehydrogenation reaction within individual palladium nanoparticles using light-coupled transmission electron microscopy.

## Introduction

Photocatalysts harvest optical energy to drive chemical reactions. They are essential for processes ranging from water purification to water splitting, air filtration, and surgical instrument sterilization. While traditional photocatalysts are comprised of semiconductors or metal complexes and clusters, pure metallic plasmonic nanoparticles have emerged as a promising new class of photocatalysts^1–4^. The enhanced photoreactivity of plasmonic nanoparticles is attributed to their localized surface plasmon resonances (LSPR), collective oscillations of the conduction band electrons which enable strong optical absorption and scattering in the subwavelength regime, with the ability to be tuned by the size, shape, material, and the surrounding medium of the nanoparticle.

The confined electromagnetic fields, local heat generation and efficient hot carrier excitation, that accompany plasmon resonances afford several advantages for photocatalysis. For example, the strong electromagnetic fields at the surface of plasmonic nanoparticles enhance the photon flux and can promote photopolymerization, photoisomerization and enantioselective reactions^5–9^. Similarly, localized heating associated with plasmonic near-fields can increase reaction rates and potentially enable spatially dependent product formation due to nanoscale reaction-rate variations^10–13^. Finally, hot carriers generated upon plasmon decay are known to activate bond formation and/or dissociation; these hot carriers have already been used to induce chemical reactions including hydrogen dissociation on gold nanoparticles and tautomerization of porphycene molecule, which, notably, are prohibitively challenging with conventional photocatalysts^14–19^.

With chemical reactions dictated by atomic and molecular interactions at the nanoscale, examining these processes with near-atomic resolution is necessary to understand photochemical processes in depth and to improve plasmonic nanoparticles for next-generation catalysts. Indeed, important and often surprising structure-function relations have been observed with single and sub-particle plasmon catalysis measurements. Utilizing techniques such as dark-field optical spectroscopy^20^, super-resolution microscopy^21–23^, and ex-situ scanning electron microscopy^24^, researchers have monitored site-specific reaction rates and identified the reactive sites of an individual particle, indicating large particle-to-particle variability. However, to date, most experiments do not have a spatial resolution better than 15 nm. Additionally, many techniques require fluorescent tags, fluorescent products, or nanoparticle markers to infer the chemically active sites, thereby introducing external species whose presence could complicate the interpretation of the results. An improved experimental scheme would offer both nanometer-scale spatial resolution and direct real-time monitoring of reaction rates.

Here, we examine plasmon-driven intercalation reactions in-situ and in real time with sub-2nm spatial resolution. To do so, we develop an environmental electron microscopy method that allows for simultaneous optical excitation. This capability enables all the benefits of an optical microscope together with the unrivaled spatial resolution of an electron microscope. A combination of aberration-corrected imaging, diffraction, and electron energy loss spectroscopy allow us to visualize the photoreactivity of nanoparticles while correlating chemical activity with particle structure. As a model system, we explore the dehydrogenation of individual Pd nanocubes adjacent to Au nanodiscs in an antenna-reactor configuration. This configuration has been recently shown useful for inducing plasmonic fields in the non-plasmonic though chemically active metal, thus promoting chemical reactions with light^22,23^.

## Results

### Identifying distinct time-constants for reaction steps

**Fig. 1:**
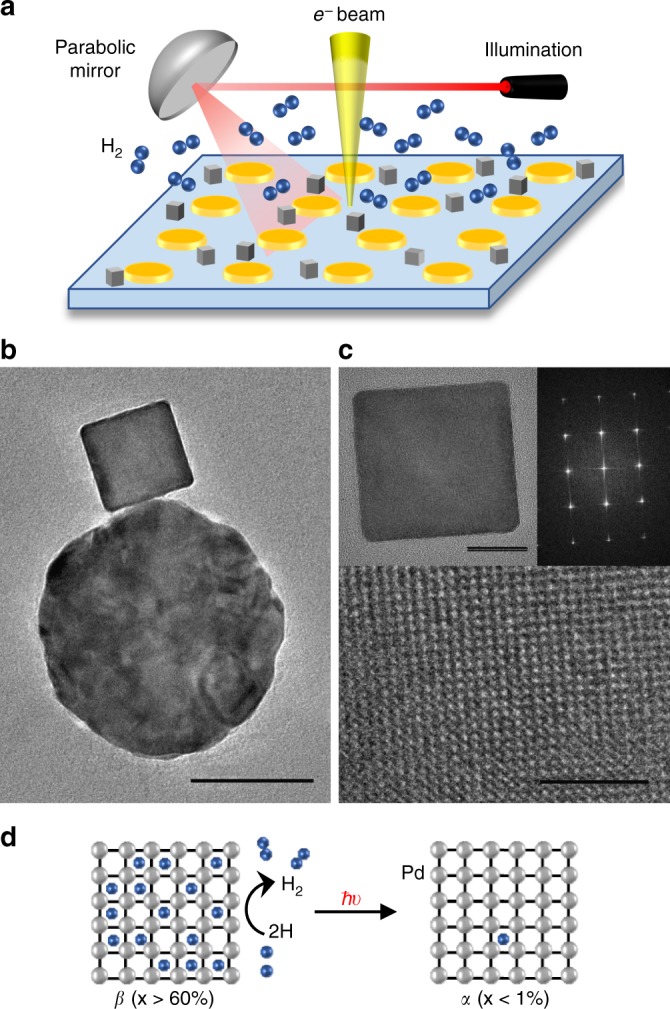
Plasmon-induced phase transformation in the antenna-reactor configuration. **a** Schematic of the light-coupled environmental transmission electron microscope (ETEM) with an Au nanodisc array and Pd nanocubes on a Si_3_N_4_ membrane. **b** Representative TEM image of an antenna-reactor pair. Scale bar is 50 nm. **c** Aberration-corrected TEM image of a representative single-crystalline Pd nanocube on an ultrathin carbon substrate, including a zoomed-in view of the lattice and its corresponding Fourier transform. Scale bars are 10 nm and 2 nm respectively. **d** Schematic of the *β* to *α* phase transformation reaction under study

Antenna-reactor pairs are fabricated in a two-step process. First, we use electron beam lithography to fabricate Au nanodiscs (the antennas), with diameters of 100–120 nm, on an electron-transparent Si_3_N_4_ membrane. Subsequently, we prepare single-crystalline palladium nanocubes (the reactors) with edge length of 30–50 nm using an aqueous colloidal synthesis. Finally, the nanocubes are drop-casted onto the patterned Si_3_N_4_ membrane to form antenna-reactor pairs. The sample is then mounted on a cryo-cathodoluminescence (CL) holder which allows us to illuminate the sample while imaging it in an environmental TEM (ETEM). The cryo-CL holder was designed by us in cooperation with Gatan, Inc. and consists of two parabolic mirrors in a compact configuration to fit the 5 mm pole piece gap of the microscope. The mirrors surround the sample and include a 500 μm wide aperture to allow the primary electron beam to pass while light is focused to/from the sample. Two optical fibers inserted into the holder enable both illumination and collection of light. Figure 1a–c shows a schematic of the experimental setup and a micrograph of a single-crystalline Pd cube adjacent to an Au nanoantenna.

We study the photocatalytic phase transformation of Pd from the hydrogen-rich *β* phase to the hydrogen-poor *α* phase, as schematically depicted in Fig. 1d. This phase transition is accompanied by a ~3.5% contraction in lattice constant and a ~2 eV red shift in the bulk plasmon resonance, which can be readily observed via diffraction and electron energy loss spectroscopy (EELS)^25,26^. We set the temperature to 243 K and start by increasing the H_2_ pressure to allow the Pd nanocube to change its phase from *α* to *β*. After the new hydrogen-rich *β* phase is confirmed, we gradually decrease the pressure to a setpoint just above the natural unloading pressure level and wait ~30 min to allow the system to reach equilibrium (see Methods for details). Depending on the particle size, the unloading pressure of our Pd nanocubes ranges from 20–40 Pa^27^. We have optimized the electron-beam dose to ensure that it is minimally-perturbative such that the nanocubes remain stably in the *β* phase without optical illumination.

**Fig. 2:**
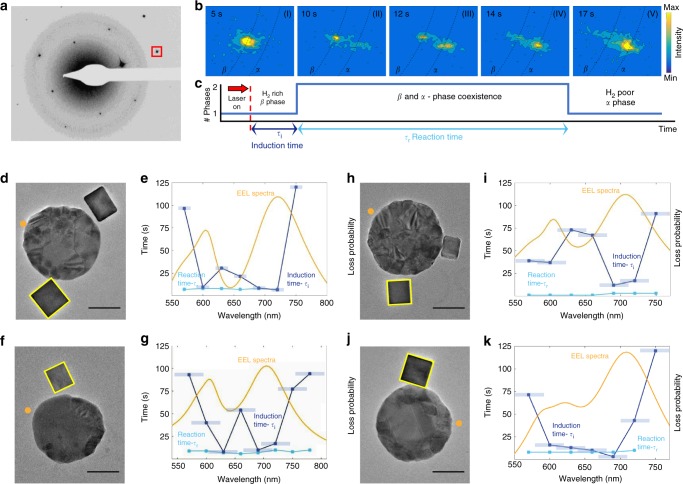
Wavelength dependence of the two reaction steps. **a** An electron diffraction micrograph of a single Pd nanocube shown in **f**. The highlighted 400 diffraction spot is monitored for the analysis of the evolution of the phase transformation reaction shown in **b**. **b** Time snapshots of a zoomed-in diffraction spot showing the emerging *α* phase and the vanishing *β* phase upon illumination. The black dotted lines correspond to the arc delineating constant lattice parameter (see Supplementary Fig. 1 as well). **c** A timeline describing the evolution of the phase transformation reaction with the two characteristic time constants, the induction time and the reaction time. **d**, **f**, **h**, **j** Transmission electron microscope images of four antenna-reactor pairs with highlighted Pd cubes for which the wavelength dependence analyses appear in **e**, **g**, **i** and **k**. Scale bars are 50 nm. **e**, **g**, **i**, **k** Wavelength dependence of the induction time constant (dark blue) and the reaction time constant (light blue). The error bars correspond the bandwidth of the laser. Simulated EEL spectra are shown in orange with an electron beam impact position marked with a dot in the corresponding TEM images

Upon illumination, the particles begin to desorb H_2_, transitioning to their α phase. The lattice contraction results in an outward shift of the single-particle diffraction points which can be easily resolved. Figure 2a, b shows the evolution of this transition upon illumination through a series of selected-area electron diffraction (SAED) time-snapshots from a single Pd nanocube. As seen, following illumination, the diffraction pattern initially remains in the *β* phase (Fig. 2bi). After several seconds, each diffraction point splits into two points, indicating *α*−*β* phase coexistence (Fig. 2bii–iv). Finally, the *β* phase point diminishes in intensity until the particle exists purely in the *α* phase (Fig. 2bv). These results indicate that the phase transformation reaction is characterized by two distinct time constants which represent the two elementary reaction steps involved in this process: the first step is the desorption of hydrogen atoms from the surface of the Pd cube; its characteristic time constant, the induction time *τ*_i_, corresponds to the time required for the system to establish a stable new phase nucleus following illumination. The second step starts immediately after the formation of the new phase and involves the continuous diffusion of hydrogen atoms from the core of the palladium lattice; its characteristic time constant, *τ*_r_, corresponds to the phase transformation reaction time (Fig. 2c and Supplementary Note 1).

The wavelength dependency of the time constants reveals a distinct difference between the behavior of the two, as can be seen in Fig. 2d–k. While *τ*_r_, the reaction time, is constant, the induction time, *τ*_i_, is highly dependent on the excitation wavelength and exhibits two dips (corresponding to enhanced reaction rates) at 610 nm and 690 nm. The dip at 690 nm is attributed to the excitation of the bright dipole plasmon mode of the gold nanodisc (the antenna)^28^. The dip at 610 nm originates from a Fano-like hybridization of dark and bright plasmonic modes mediated by the substrate, enabling far-field coupling^29,30^. Both of these modes are apparent in our scanning-TEM-EELS measurements, as well as in our full-field simulations (Supplementary Fig. 2-4). Note that in control experiments, without the Au nanodiscs antennas, the Pd nanocubes remain loaded and show no response to the external illumination (Supplementary Fig. 5 and Supplementary Note 2). Accordingly, the wavelength-dependency we see arises purely from a plasmonic response.

The distinct wavelength dependence of the induction and reaction time constants suggests that the desorption of hydrogen from the Pd surface has a higher activation energy than the second step of the reaction, which is associated with hydrogen diffusion towards the surface and the accompanying lattice rearrangement (Supplementary Note 3). This difference in activation barriers is also found in bulk measurements with activation barriers of ~5–20 kcal∙mol(H_2_)^−1^ ^31,32^ and ~5 kcal·mol(H_2_)^−1^^33^ for the desorption and the diffusion processes respectively.

### Mapping site-selectivity at plasmonic hot-spots

**Fig. 3:**
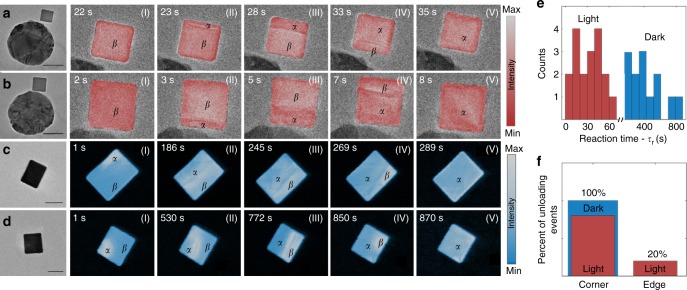
Comparison between dark and light-induced phase transformation reaction. **a**–**d** Snapshots of (scanning) transmission electron microscope movies of the phase transformation reaction in the antenna-reactor (isolated) configuration with(out) illumination at 690 nm. Scale bars are 50 nm. **e** Histograms showing the distribution of reaction times for the plasmon-induced phase transformation (red) and the spontaneous phase transformation (blue) as measured in STEM mode. **f** Corner vs. edge unloading mechanism with and without illumination, also measured in STEM mode

While SAED allows us to monitor the progression of the photocatalytic phase transformation of individual nanoparticles, it does not capture the spatial response of the system. Using STEM and TEM imaging we can map the progression of the phases in real-time with ~2 nm spatial resolution; concurrently, we use STEM-EELS to assign regions to a particular phase. Figure 3a–d shows how the phase transformation evolves through a series of time snapshots for two antenna-reactor pairs under illumination at 690 nm (Fig. 3a, b) and for two isolated Pd nanocubes under dark conditions (Fig. 3c, d) at a given pressure. As seen, the plasmon-induced phase transformation begins with the formation of an *α* phase nucleus at one edge of the particle. A (100) phase front is formed and propagates across the particle until the *β* phase is completely pushed out of the particle, again through an edge of the cube. Depending on the imaging conditions, the average reaction time, *τ*_r_, is ~10 s for TEM imaging (measured for 21 particles in 90 unloading events) and ~30 s for STEM imaging (measured for additional 7 particles in 18 unloading events).

Such results are remarkably distinct from the transformation that occurs in the dark. Here, we induce phase transformation by lowering the hydrogen pressure below the thermodynamic unloading pressure of the nanocubes. As seen, the reaction begins with the formation of an α phase nucleus in the corner of the particle (i.e the lowest- coordination number site), showing a phase boundary which is consistent with a (111)-interface. After nucleating, the *α *phase grows while the phase front reorients until stabilizing along the <100> direction. The *α* phase front then propagates until the *β* phase is restricted to a corner of the particle. Again, a (111)-like interface is formed, in contrast to the plasmon-mediated transformation. Interestingly, this dark phase transformation reaction is more than 10 times slower compared to the light induced one, with an average reaction time of ~400 s (Fig. 3e). Additionally, while phase nucleation from the corner of the cube is always observed in the dark (total of 16 unloading events), optical illumination appears to enable edge nucleation as observed in 20% of the STEM measurements (total of 15 unloading events) (Fig. 3f) and in 50% of the TEM measurements (total of 20 additional unloading events); Measurements of antenna-rector pairs in the dark also showed corner nucleation, confirming that edge nucleation is not facilitated by the presence of the antenna itself (Supplementary Fig. 6). An increased reaction rate that is beyond our temporal detection resolution could explain these observations, but it is also possible that plasmons are modifying the reaction mechanism. In particular, plasmons could lower the activation barrier for hydrogen diffusion through different crystallographic orientations, making the lowest coordination number sites not necessarily the most chemically reactive.

**Fig. 4:**
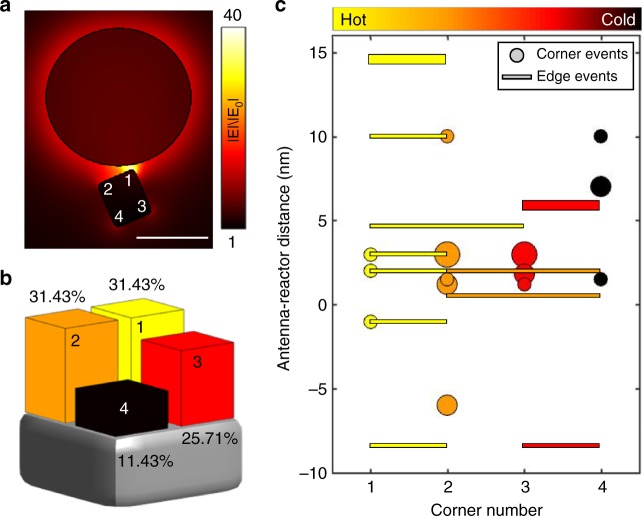
The effect of electromagnetic hot spots on the phase transformation reaction. **a** Calculated field enhancement for the antenna (Au) – reactor (Pd) system under planewave excitation at 690 nm. The corners of the Pd nanocube are labeled 1-4 with 1 being the corner closest to the hot spot and 4 the farthest. Scale bar is 50 nm. **b** Distribution of plasmon-induced phase transformation events by corner number. **c** Distribution of plasmon-induced phase transformation events with respect to the distance between the nanodisc (antenna) and the nanocube (reactor). Negative antenna-reactor distance values correspond to a physical overlap between the nanodisc and the nanocube. The size of the markers corresponds to a single unloading event (small markers), 2 unloading events (medium markers) and 3 unloading events (big markers). Edge events are marked with bars spanning the two corners comprising the edge. For the summary analysis of **b**, we assign each edge unloading event to one corner number (the lower corner number)

In addition to modifying reaction rates and mechanisms, plasmons can also enable site-selectivity through their localized strong electromagnetic fields, known as hot spots. Such site-selectivity has been observed in other recent single particle catalysis studies utilizing super-resolution microscopy^21,23^ and scanning electron microscopy^24^. To investigate site-selectivity in Pd dehydrogenation we correlated real time images of the unloading reaction with simulated local electromagnetic field enhancements in antenna-reactor pairs of varying separation and orientation. As seen in Fig. 4a, the electromagnetic field is highly concentrated at the surface of the Au nanodisc with a hot spot in the gap between the Au nanodisc and the Pd nanocube. We analyzed 35 phase transformation reactions in an antenna-reactor configuration and for each we labeled the cube’s corners to reflect the relative distance from the hot spot (corner 1 is the closest and corner 4 is the farthest). As seen in Fig. 4b, upon illumination, over 31% of the particles nucleate the α phase at a corner adjacent to the hot spot while only 11.4% of the particles nucleate at the farthest corner. Without illumination, nucleation is random, and all corners desorb with equal probability (~25%).

## Discussion

Plasmons can mediate chemical transformations either by enhanced light absorption, excitation of hot carriers, or by local induced heating resulting from nonradiative plasmon decay. In the antenna-reactor configuration, these phenomena can manifest themselves in either the antenna or in the reactor itself due to near-field coupling which induces a forced plasmon in the non-plasmonic material^34,35^. As seen in Supplementary Fig. 7, coupling in the antenna-reactor system leads to a visible-frequency absorption cross section of the Pd nanocube that rivals that of the Au nanodisc. First, using these absorption cross-sections, we can model the temperature of the antenna-reactor system under our experimental illumination conditions. Time-dependent simulations show a time-average increase of ~1 K for the Au nanodisc and ~7 K for the Pd nanocube. While instantaneous temperatures peak at 340 K and 640 K for the Au nanodisc and Pd nanocube, respectively, such temperatures decay back to steady-state values within 200–400 ps. Due to the Pd particle’s small size and fast thermal diffusion (thermal diffusivity of ~10^−^^5^ m^2^/s), a temperature gradient across the Pd nanoparticle is unlikely to form (Supplementary Note 4). Thus, while heating contributes to the enhanced kinetics of the transformation, temperature alone cannot account for the observed site-selectivity.

Photogenerated carriers can also contribute to the reaction, either through direct excitation of the molecular orbitals or through indirect excitation via hot carriers. In the latter case, hot carriers from the metal populate the antibonding adsorbate orbitals, leading to a significant displacement from the equilibrium of the adsorbate geometry as well as to a different energetic profile. As recently shown for photodesorption^36,37^ and photoadsorption^15^, the return of the system to its ground electronic state is accompanied by significant energy redistribution into translational, vibrational and rotational degrees of freedom, and consequently, destruction of the chemical bond. In bulk palladium hydrides, hybridization between the atomic orbitals of Pd and H leads to the formation of a bonding orbital with energy of −6.5 eV with respect to the Pd fermi level and another orbital with an antibonding characteristic emerging at 2.5 eV above the fermi level^38–40^. These energies could shift with nanostructuring, but given the illumination energies used in our experiment (1.6–2.1 eV), we hypothesize that indirect charge transfer and the formation of a transient ion are not the dominant contributions to the phase transformation reaction.

On the other hand, direct absorption within the hybridized Pd–H molecular orbitals, which does not involve charge transfer from the metal, is more likely to contribute to the enhanced reaction rate and site-selectivity. In this case, population of the hybridized Pd and H atom orbitals brings the system to an excited state and results in smaller activation barriers along the reaction pathway compared to the ground state (Supplementary Fig. 8)^41^. Therefore, the increased absorption cross-section throughout the Pd cube expedites the phase transformation. Additionally, regions of highest absorption on the surface of the Pd nanocube, such as the hotspot, are more likely to get excited, thus promoting site-selectivity.

Since this mechanism depends on local absorption in the Pd nanocube, it is important to examine site-selectivity with respect to the antenna-reactor distance which will govern its efficiency. Figure 4c presents such an analysis: at overlapping antenna-reactor separations, where there is a physical contact between the Pd nanocube and the Au antenna, none of the unloading events nucleate at the cold corner, number 4. At longer antenna-reactor distances, near field coupling with the Au antenna is less effective, leading to a smaller absorption cross section with less variation between different sites on the nanocube. In these cases, all four corners of the cube will act as local hot spots, with corner number 1 remaining the hottest due to its proximity to the antenna. In contrast to the overlapping regime, where none of the studied phase transformations began at corner number 4, at larger antenna-reactor separations ~14% of the cases show unloading from this cold corner. These results emphasize the capability of plasmons to control chemical reactions through the formation of local hot spots and highlight the future potential of enabling site-specific reactions at the nanoscale.

In summary, we have presented an experimental method which allows us to observe and control photocatalytic reactions with sub-2nm spatial resolution spatial resolution. Using a light-coupled environmental TEM, we are able to follow in situ and in real time the plasmon-induced dehydrogenation of individual palladium nanocubes. The *β* to *α* phase transformation reaction is comprised of two elementary steps with different activation barriers, such that the excitation of plasmons affects the rate of each step differently. Under illumination, the reaction rate is enhanced by a factor of 10 compared to ambient conditions. Moreover, we find that plasmons appear to modify the reaction mechanism, within our time-resolution, allowing the *α* phase to nucleate at an edge of the cube instead of at the corners in a statistically significant number of particles. Our single particle measurements also allow us to correlate the active sites of the Pd nanocube with the formation of plasmonic hot spots, suggesting that dehydrogenation is expedited due to locally-enhanced photo-absorption and direct excitation of Pd–H molecular orbitals. Looking forward, optical excitation and detection with an environmental TEM holds promise to explore the influence of particle structure on photocatalytic behavior for a range of reactions beyond phase transformations. For example, Raman spectroscopy and high-resolution electron-energy loss spectroscopy can be used to follow a variety of industrially-relevant reactions in the environmental TEM. As the community continues to gain better control over the variety of plasmon decay pathways there is significant promise for high-efficiency, site-specific, and product-selective photocatalysis.

## Methods

### Gold nanodiscs fabrication

Au nanodiscs are fabricated on a 30 nm thick, 100 μm wide single window, silicon nitride (Si_3_N_4_) membrane (SPI Supplies) using the following process: First, we spin coat polymethyl methacrylate (PMMA) 495k molecular weight 4% in anisole (MicroChem Corp.) onto the membrane at 4500 rpm for 40 s. After baking at 180 °C for 3 min, the PMMA is exposed in a JEOL JBX-6300FS EBL system, followed by 1 min development in a 3:1 isopropyl alcohol (IPA): methyl isobutyl ketone (MIBK) solution. Then, we deposit a 1 nm adhesive layer of Ti and 30 nm of Au in KJ Lesker e-beam evaporator. After lift-off in acetone, an array of Au nanodiscs with diameter of 100–120 nm and pitch of 1 µm is obtained (Supplementary Fig. 9).

### Palladium nanocube synthesis

Single-crystalline {100}-terminated Pd nanocubes were synthesized following a previously reported method^25^. The particles were stabilized with hexadecyltrimethylammonium bromide (CTAB) and were washed twice with water to remove excess ligands, before drop-casted on the Si_3_N_4_ membrane.

### Microscopy

The Si_3_N_4_ membrane with the antenna-reactor nanoparticles is cleaned for one minute in an Ar/O_2_ plasma at 50 W of RF power and then mounted on a TEM cathodoluminescence (CL)-cryogenic holder (Gatan, Inc.) that in addition to illuminating the sample, controls the temperature to ± 0.1 K. To avoid condensation of contaminants on the sample during hydrogen gas flow, we use a liquid nitrogen cooled cold finger which minimizes beam-induced hydrocarbon contamination during data acquisition at all hydrogen pressures. All experiments are carried out using an FEI Titan 80–300 environmental (scanning) transmission electron microscope operated at 80 kV for the STEM experiments and 300 kV for high-resolution imaging and diffraction as described later. The microscope is equipped with a monochromator, an aberration corrector in the image forming objective lens and a Gatan 966 Quantum electron energy loss spectrometer. The H_2_ (99.9999%, Praxair) pressure can be varied between 4 and ∼600 Pa using a home-built mass flow controller operated gas manifold system.

### TEM phase transformation experiments under illumination

Unpolarized light emitted from a supercontinuum laser (SuperK Extreme, 78 MHz, ~100 ps pulse, NKT Photonics) is guided to the CL holder through an optical fiber (multimode, Thorlabs) and focused onto the sample by the holder-integrated parabolic mirror to form a spot size of 200 μm in diameter. The bandwidth of the laser pulse is kept constant throughout the experiment and set between ±10 nm to ±25 nm corresponding to power levels of 1.5±0.3 mW and 3.8±0.3 mW respectively, which are measured before the entrance point to the TEM. This setting depends on the amount of power required to induce the *β* to *α* phase transition reaction which is derived directly from the operating H_2_ pressure and its level above the pressure characterizing the nanocube’s spontaneous phase transition. The diffraction patterns were obtained at 300 kV using a 180 nm selected area aperture where electron dose rate is 5 electrons/Å^2^ sec. At the beginning of the experiment, the sample is heated to 320 K in 500 Pa of H_2_ for 30 min, to reduce any oxides formed during the plasma treatment. Then, the sample is cooled to 243 K to allow the uptake of hydrogen by the Pd nanocube and the system is let to equilibrate for 30 min. The new hydrogen-rich *β* phase of the Pd nanocube is then confirmed by comparing the selected area electron diffraction (SAED) pattern of the single particle to its original state and the pressure is reduced to <4 Pa until the nanocube is unloaded again and returns to its *α* phase. This hydrogenation and dehydrogenation cycle is repeated twice in order to remove any residual strain buildup and stabilize the particle^25^. Next, in order to identify the pressure level at which the Pd nanocube unloads spontaneously, we acquire a pressure-composition unloading isotherm with no illumination (Supplementary Fig. 10). We start by increasing the H_2_ pressure until the particle is fully loaded again, and continue by collecting a series of SAED patterns at different descending pressure points, until the particle is fully unloaded and in its hydrogen-poor, *α* phase. At each pressure point we wait ~30 min for the system to reach equilibrium before collecting the diffraction patterns. The separations between pairs of spots in the diffraction pattern are represented as percentage changes from the separation in the reference pattern of the fully loaded state. Finally, for the plasmon-induced reaction, the H_2_ pressure is increased to allow the particle to load once again, then we decrease the pressure to a point just above the spontaneous unloading level (~+20 Pa). After the system has reached equilibrium (~30 min), and before illuminating the sample, the *β* phase of the particle is verified again to ensure its initial state. The laser is then turned on and diffraction video is recorded for 2 min with temporal resolution of 4 frames/sec. We repeat this experiment for different wavelengths while keeping the power and the H_2_ pressure constant.

### Live monitoring of the phase transformation using STEM

The sample was first heated to 333 K at hydrogen pressure of 473 Pa for 30 min, then cooled to 238 K, which was set constant as the operating temperature for the live-STEM monitoring of the phase transformation. The particles were cycled (hydrogenated and dehydrogenated) once before collecting any data. The bulk plasmon mode of Pd shifts from 8 eV to 6 eV upon dehydrogenation thus allowing us to use EELS to determine the phase of the nanocube. After making sure the particles are hydrogenated, we slowly reduced the pressure to 0 Pa and started monitoring particles. The monochromator was excited in the STEM mode with monochromator spot size of 15 and STEM beam size of 9. The scan time per pixel was kept constant at 4 μs. The magnification was also kept constant for imaging all particles (640kX) to ensure similar beam dosage. The annular dark field (ADF) detector was used with a small camera length of 48 mm, for which the collection angles are between 55 and 70 mrad. Under these conditions, the ADF detector collects only some portions of the diffraction disks from 400, 600, and 620 reflections for <100> zone axis. With dehydrogenation, strain and/or defects in the lattice, as well as the change in lattice parameter itself, result in imaging contrast as observed in the STEM scans. During the slow phase transformation, we momentarily paused the scanning to collect EEL spectrum from different contrast regions to confirm the phase at each point. The EEL spectra were collected for 0.05 s. The movies were recorded using a screen-recording software (CamStudio), and then processed in MATLAB to enhance contrast.

### Simulations

Simulations have been performed by using the boundary element method (BEM) implemented using Matlab toolbox, MNPBEM^42^. The optical constants of Au have been taken from Johnson and Christy^43^ and of PdH from Duine^44^. The Si_3_N_4_ membrane was modeled with refractive index of 2.05.

## Electronic supplementary material

Supplementary Information

## Data Availability

All relevant data are available from the corresponding authors upon request.
