# Supplementary material for: In-situ observation of plasmon-controlled photocatalytic dehydrogenation of individual palladium nanoparticles
Source: Nat Commun. 2018 Nov 7;9:4658. doi: 10.1038/s41467-018-07108-x (PMC6220256; doi:10.1038/s41467-018-07108-x)
Supplement: Supplementary file 1 — Supplementary Information [file 41467_2018_7108_MOESM1_ESM.pdf]

In-situ observation of plasmon-controlled photocatalytic dehydrogenation of individual palladium nanoparticles

Vadai et al.

Supplementary Figure 1

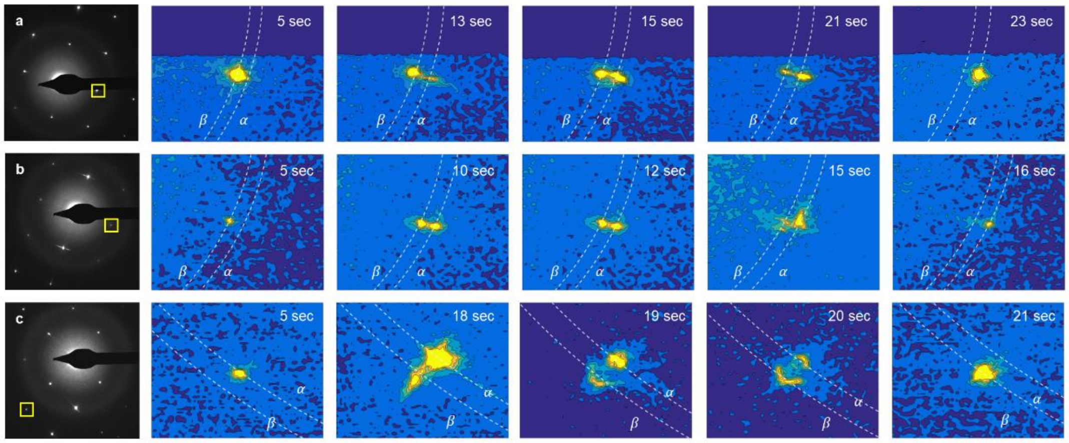

Time snapshots extracted from electron diffraction movies. a-c Time snapshots of a zoomed-in diffraction spot (a,b – (200), c- (400)) showing the emerging α phase and the vanishing β phase upon illumination at 690 nm corresponding to the on-resonance phase transformation reaction of the nanocubes shown in Figure 2 (d, j, h). The white dotted lines correspond to the arc delineating constant lattice parameter.

Supplementary Figure 2

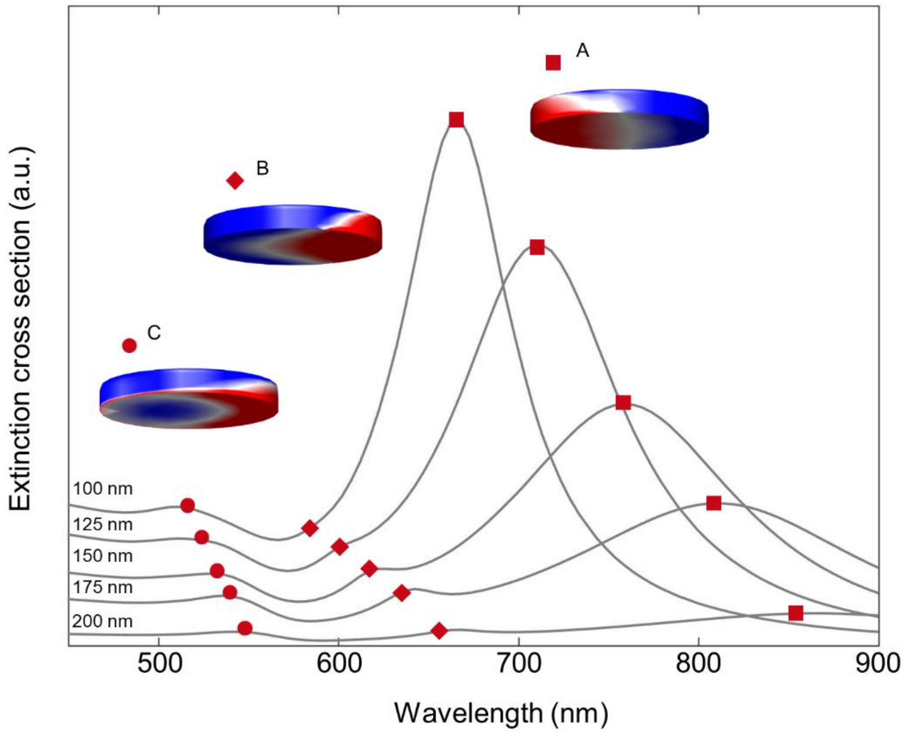

Simulated extinction cross section of an Au nanodisc. BEM simulations of the extinction cross section of a single Au nanodisc with varying diameter, on a 30 nm thick Si_3N_4 substrate. The spectra show 3 distinct bright modes (A-C) with finite dipole moments as indicated by the corresponding charge maps. The presented spectra are simulated with a TM planewave source with an angle of incident of 40 degrees with respect to the normal to the surface.

Supplementary Figure 3

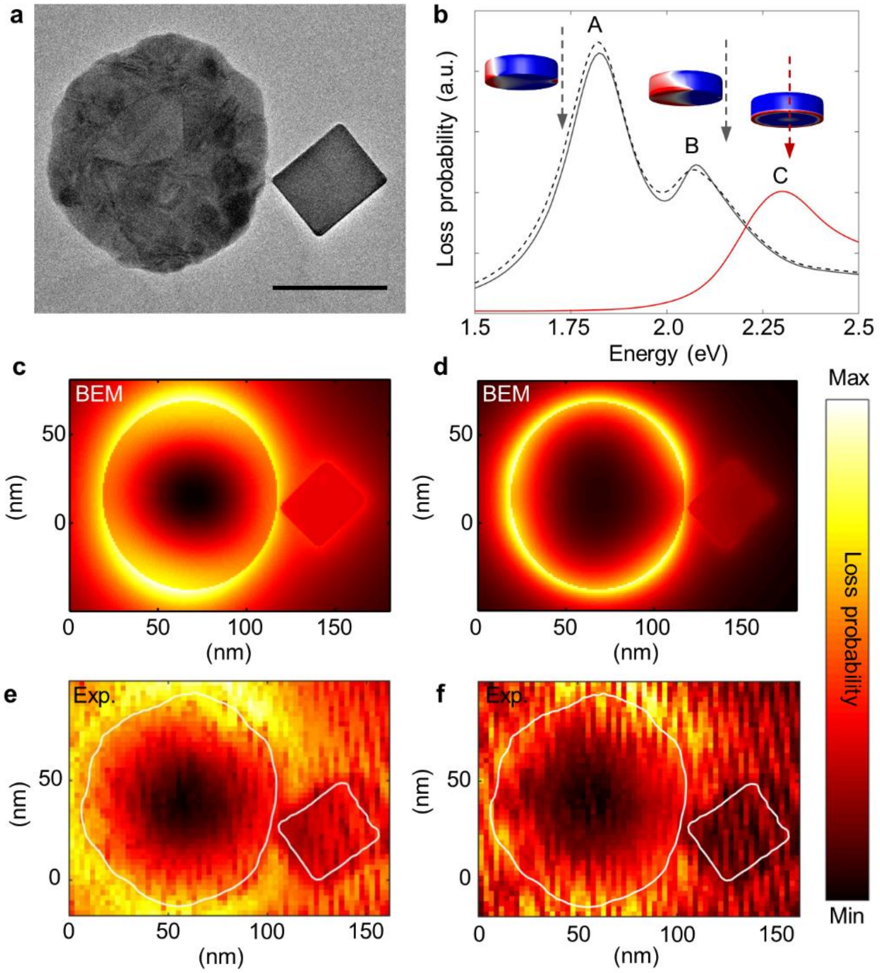

Electron energy loss spectra of the reactor-antenna pair. a, Transmission electron microscopy image of the antenna-reactor pair. Scale bar is 50 nm. **b,** Simulated EEL spectra of an Au nanodisc, 100 nm in diameter, on a Si_3N_4 substrate, showing the 3 plamon resonances (A-C) and the associated charge maps. For modes A and B, the spectrum (black curve) was simulated by positioning the e^- beam 5 nm away from the edge of the nanodisc (indicated by the arrow) while for mode C the e^- beam was positioned at the center of the nanodisc (red curve). The EEL spectrum for the full antenna-reactor system including the Pd nanocube is similar to the one obtained for the nanodisc alone (dashed black line). **c-f,** Simulated and experimental EELS maps at energies of 1.82 eV (**c**, **e**) and 2.08 eV (**d** and **f**) corresponding to modes A and B in (**b**).

Supplementary Figure 4

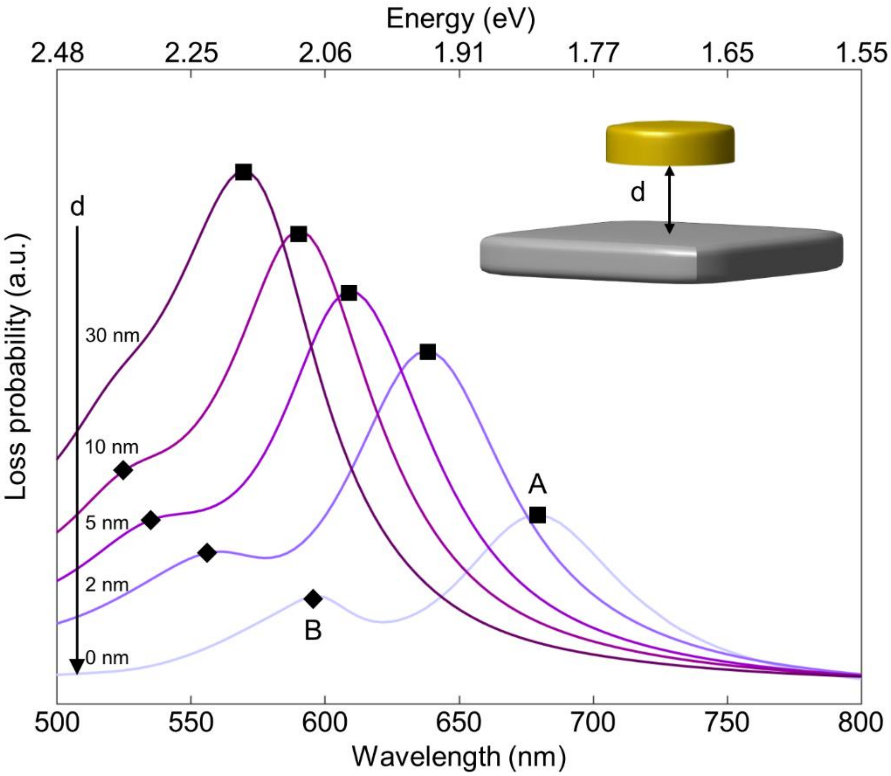

Substrate-mediated Fano resonance. Simulated EEL spectra of an Au nanodisc with varying distance to the underlying Si_3N_4 substrate. The hybridization between the two nearly degenerate modes becomes more pronounced as the nanodisc approaches the substrate. The two plasmon resonances, A and B, are easily resolved when the nanodisc touches the substrate with no separation in between.

Supplementary Figure 5

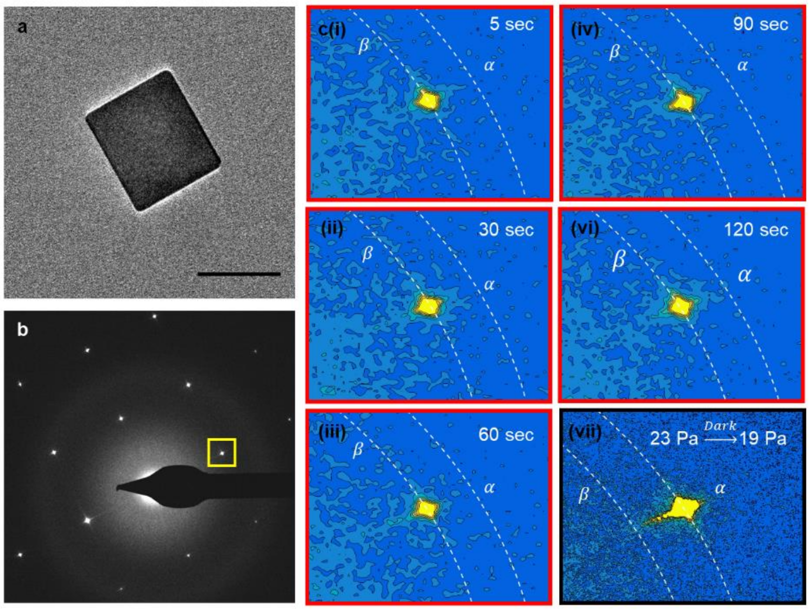

Control experiment without Au nanodiscs. (a-b), Representative Pd nanocube on a 30 nm thick Si_3N_4 membrane and its corresponding electron diffraction micrograph. Scale bar is 20 nm. **c(i)-(vi)**. Sequential images extracted from control experiment, attempting to induce phase transformation using light without the presence of the Au nanodiscs. The highlighted 200 diffraction point in (b) is monitored for 2 min while the pressure is kept constant at 23 Pa and the sample is constantly illuminated at 690 nm with power of 4 mW. **(vii)**. After 2 minutes without any detectable phase transformation, the reaction is induced in the dark by lowering the pressure to 19 Pa. This step confirms that the excitation of plasmons within the Au nanodiscs is imperative for the phase transformation reaction and light by itself doesn't promote the reaction even at lower pressure, very close to the natural unloading pressure of the Pd nanocube.

Supplementary Figure 6

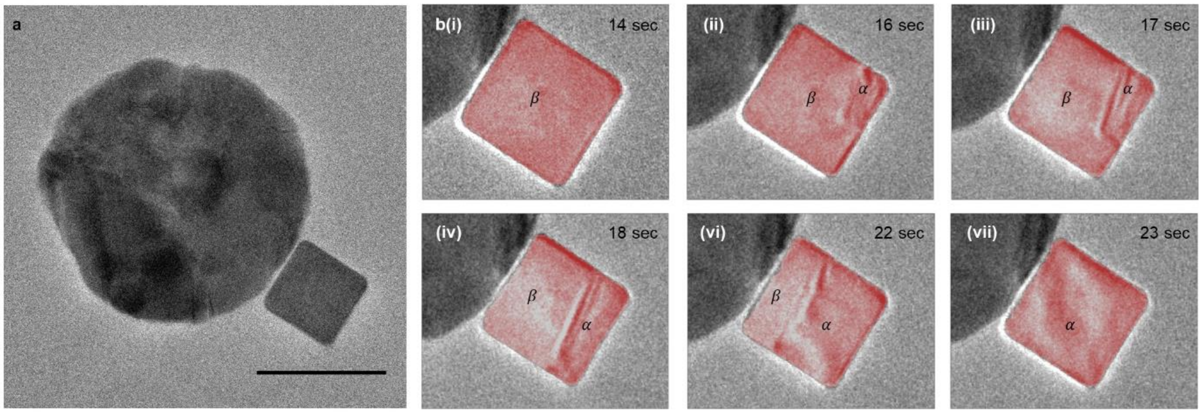

Control experiment without illumination. a, Transmission electron microscopy image of the antenna-reactor pair. Scale bar is 50 nm. **b(i)-(vii),** Snapshots of transmission electron microscope movies of the phase transformation reaction in the antenna-reactor induced by lowering the hydrogen pressure to 17 Pa without illumination. The electron dose rate used in this experiment was higher than the usual 5 electrons/Å^2 sec rate reported for all other experiments, thus the comparison of the reaction kinetics is irrelevant. Nevertheless, this experiment shows that edge nucleation is not facilitated just by the presence of the antenna itself.

Supplementary Figure 7

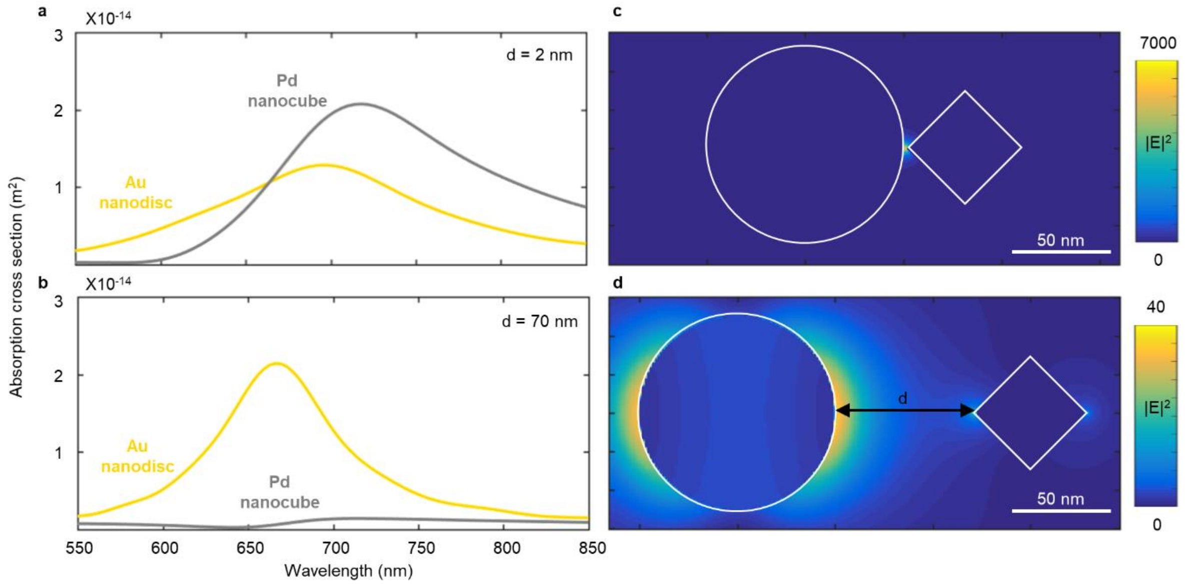

FDTD simulated absorption cross sections and field enhancement. (**a,b**) Absorption cross section for the Pd nanocube and Au nanodisc with interparticle distance of d=2 nm and d =70 nm. (**c,d**) Electromagnetic field distribution maps for the corresponding geometries.

Supplementary Figure 8

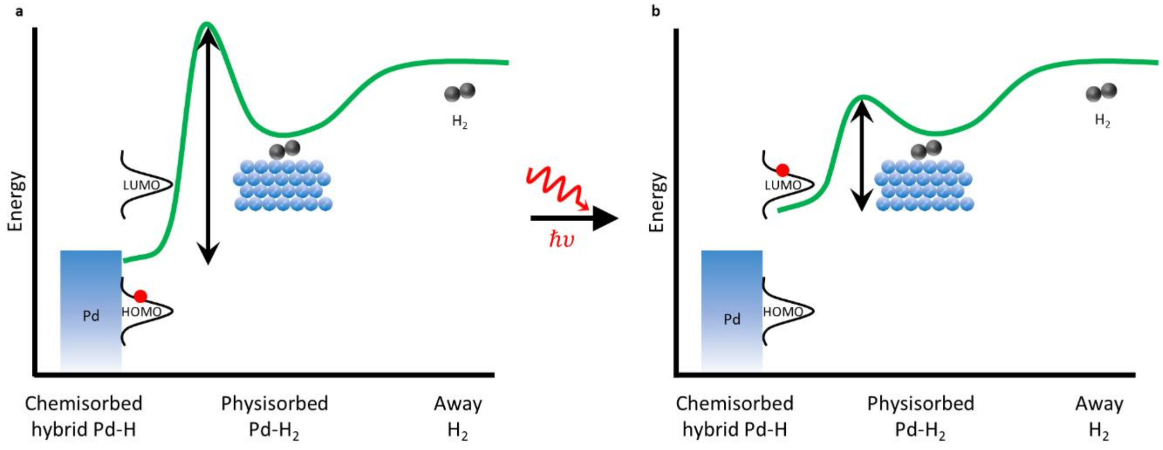

Ground state and excited state reaction pathways. Direct absorption in the Pd nanocube, due to near field coupling with the Au antenna, populates hybridized Pd-H orbitals, bringing the system into an excited state (**b**). This process provides enough energy to overcome smaller activation barriers on the reaction pathway compared to the larger barriers existing in the ground state (**a**).

Supplementary Figure 9

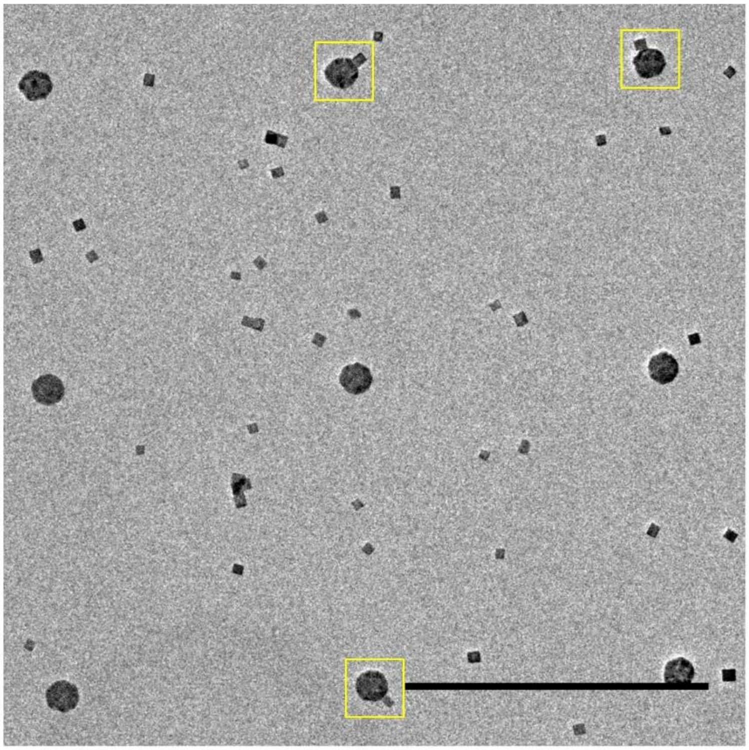

Representative sample. Transmission electron microscopy image of a representative sample used in this study, with three antenna-reactor pairs highlighted in yellow. Scale bar is 1 μm.

Supplementary Figure 10

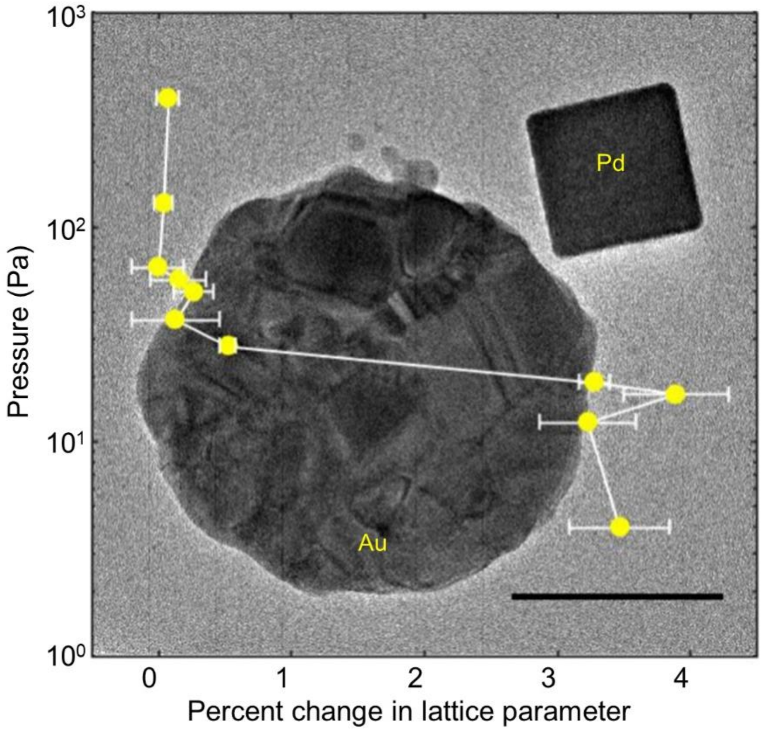

Hydrogen unloading isotherm. Pressure-lattice constant isotherm collected without illumination using electron diffraction for a single Pd nanocube (background TEM image) measured at 243 K. The percent change in lattice parameter corresponds to a lattice contraction. The error bars correspond to the standard error of distribution of the changes in diffraction points separation. Scale bar is 50 nm.

Supplementary Figure 11

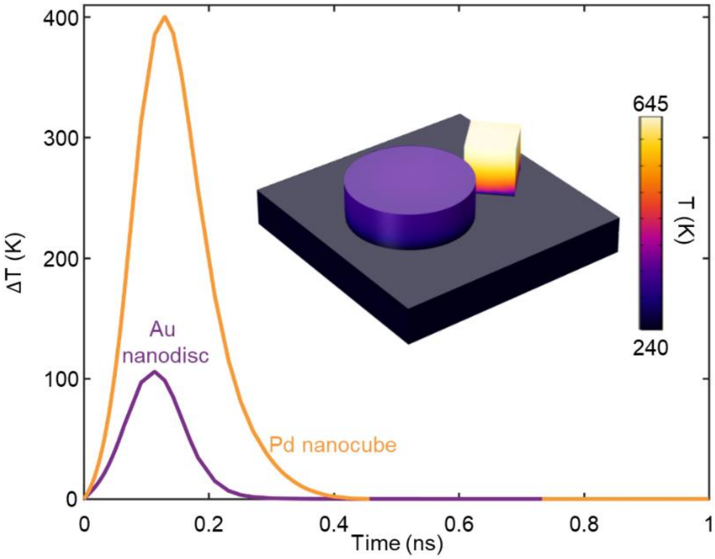

Transient temperature increase in the Au nanodisc – Pd nanocube system upon pulsed illumination. Temperature increase reaches it maximum value after ~ 130 ps after which quick relaxation takes place that brings both particles to their steady-state temperature of 240 K. Inset shows the temperature profile in the system after 130 ps. For the simulation the absorption cross sections for the Au nanodisc (100 nm diameter, 30 nm height) and for the Pd nanodisce (40 nm edge length) were taken from the FDTD simulations presented in Supplementary Figure 7 at illumination wavelength of 700 nm.

Supplementary Figure 12

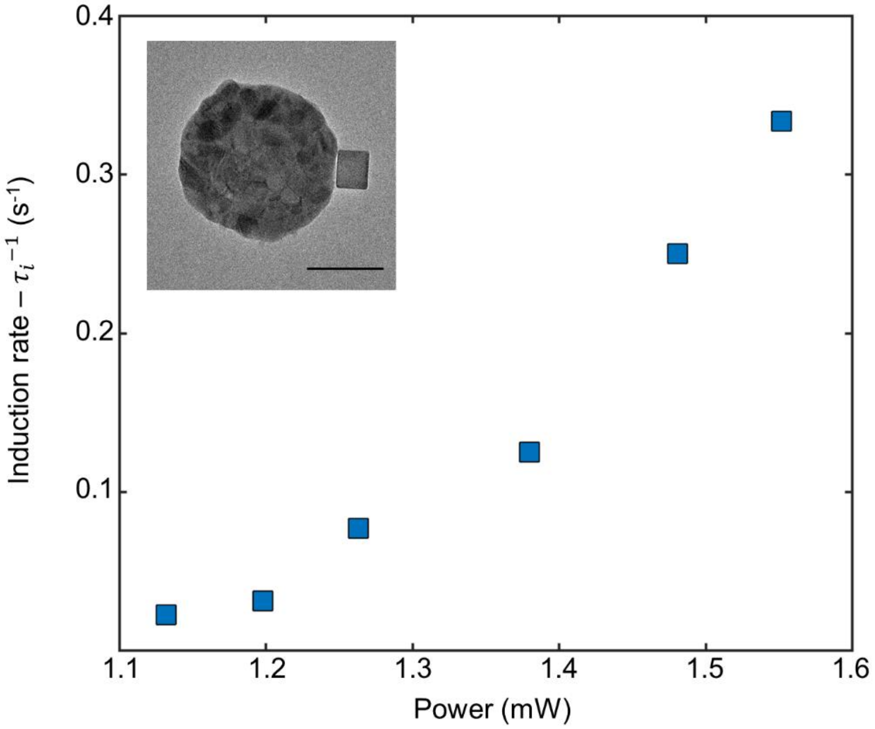

Power dependence measurement. The induction rate, τ_i^{-1}, shows a nonlinear behavior as a function of the incident power of the laser, implying involvement of multiple photoexcited charge carriers and/or thermal effect. The inset shows the antenna-reactor pair for which the phase transformation induction rate was measured. Scale bar is 50 nm.

Supplementary Note 1: The definition of 'induction time' and its relation to hydrogen desorption/diffusion from the Pd lattice

Our previous results[1–3] indicate that single-crystalline palladium nanoparticles (like the investigated nanocubes) do not sustain thermodynamic phase coexistence; the core of single-crystalline nanoparticles will exclusively be either α phase or β phase in equilibrium. This result is evidenced by both EELS, allowing us to assign each region in the nanocube to a specific phase, as well as diffraction. Using both techniques, sharp transitions in pressure-composition isotherms are obtained. Also using both techniques, we have shown that both in the dark and upon illumination, the transformation reaction is initiated by the nucleation of the new phase at the corner of the cube. Naturally, there must be a vacant site in the palladium lattice to host the hydrogen atom while it diffuses out of the nanocube. However, to date, our method is not sensitive to the sub-surface, and we cannot rule out diffusion of hydrogen from the ~1 nm subsurface of the palladium cube. It is possible that hydrogen from the first few atomic monolayers of the Pd cube diffuses out before we are able to observe a change in the particle. Thus, we included this uncertainty in the definition of the induction time by explicitly specifying that we refer to the *observed* nucleation of the new phase as the onset for the diffusion of hydrogen atoms and the beginning of the second reaction step. Nevertheless, the fact that our measurements allow us to distinguish between two subsequent steps with different wavelength dependences, (the induction time and reaction time) implies that each of these steps is governed by one primary mechanism, characterized by a different activation energy.

Supplementary Note 2: The role of Au nanodiscs

The presence of the Au nanodiscs next to the Pd nanocubes is important in two aspects: first, it significantly improves the visible-frequency absorption cross section of the Pd nanocube by inducing a "forced plasmon"; second, it breaks the symmetry of the geometry, compared to an isolated Pd nanocube, by forming a plasmonic hot-spot in the nanogap. This makes the corner of the Pd nanocube closest to the Au antenna more reactive and eventually leads to the observed site-selectivity. Nevertheless, we would expect that illuminating an isolated Pd nanocube in the UV, nearer to the plasmon resonance of the Pd itself, would also affect the kinetics and possibly the mechanism (for example, by illuminating at the corner versus edge mode of the cube). In that case however, no site-selectivity is expected to be seen and a much higher illumination power is

needed since the absorption cross section for the Pd itself is relatively poor. Such a study is beyond the scope of this paper and is the subject of future work.

Supplementary Note 3: Near-constant reaction time with wavelength

The diffusion of hydrogen in the lattice has a lower activation energy than the desorption of hydrogen from the surface. While the excitation of plasmons in the Au-Pd system overcomes the activation barrier for the diffusion even in 'off resonance' wavelengths, it is still insufficient to bring the system to a higher energetic state beyond the activation barrier for the desorption of hydrogen. Basically, the excitation of plasmons at different wavelengths modifies the 'effective' activation barrier for the desorption process while it completely removes the activation barrier for the hydrogen diffusion step. The difference between the two activation barriers with the one for diffusion being lower than that for the desorption is also found in bulk measurements of palladium hydrides. It is worth pointing out that lower laser intensities and further off-resonant wavelengths that we have tried using weren't sufficient enough to initiate the reaction by inducing the desorption of hydrogen from the surface and thus prevented the second reaction step from occurring.

Supplementary Note 4: Temperature increase upon illumination and its contribution to the observed results

In order to estimate the temperature increase upon illumination in the antenna-reactor system, we used COMSOL to simulate the time-dependent temperature evolution and its profile due to pulsed illumination. We consider pulsed illumination with peak power of 1500 W/cm^2 (corresponding to an average power of 5 mW of a 100 ps pulse at 700 nm with repetition rate of 78 MHz). We also consider a 30-nm thick silicon nitride membrane with a thermal conductivity of 2 W/mK[4] at our operating temperature (243 K). As seen in Supplementary Fig. 11, our calculations indicate a transient temperature increase of ~100 K for the Au nanodisc and ~400 K for the Pd nanocube. The contact with the cooled membrane helps bring the two nanoparticles to their steady state temperature of 240 K within ~ 200 - 400 ps. Thus, the average temperature increase of the Au nanodisc is less than 1 K while for the Pd nanocube it is ~ 7 K. Except for localized heating, when illuminating large number of particles, it is also important to consider the collective heating of the sample in which case the temperature becomes uniform[5]. Under these conditions we cannot rule out heating effects as the reason for the enhanced kinetics, which are

likely to be convoluted with the effects emerging from hot carrier excitation. This assumption is further confirmed by power dependent measurements of the reaction rate which show nonlinear behavior of the induction rate, ${\tau_i}^{-1}$ (Supplementary Fig. 12). This nonlinear dependence can be related to thermal effects[6] as well as to multiple electronic excitations[7].

However, heating by itself cannot account for the site selectivity unless a temperature gradient across the Pd nanocube is formed. Our COMSOL simulations take into account a uniform absorption cross section ($1\cdot10^{-14}$ m^2 for the Au nanodisc and $2.5\cdot10^{-14}$ m^2 for the Pd nanodisc taken from Lumerical FDTD simulation as shown in Supplementary Figure 7) and in such case, as can be seen in Supplementary Fig. 11, the temperature is uniform across each nanoparticle. Nevertheless, a transient local temperature increase can be formed on one side of the Pd nanocube due to an enhanced local absorption at the hotspot which will result in an increased hydrogen desorption at this site. Due to fast thermal equilibration in the Pd lattice, in the order of ~ 100 ps (thermal diffusivity 10^{-5} m^2 s^{-1})[8], we assume this is not the dominant process. Thus, we can conclude that while temperature increase might play a role in enhancing the reaction rate, it cannot account for the observed site selectivity.

Supplementary References

1. Baldi, A., Narayan, T. C., Koh, A. L. & Dionne, J. A. In situ detection of hydrogen-induced phase transitions in individual palladium nanocrystals. *Nat. Mater.* **13,** 1143–1148 (2014).
2. Narayan, T. C., Baldi, A., Koh, A. L., Sinclair, R. & Dionne, J. A. Reconstructing solute-induced phase transformations within individual nanocrystals. *Nat. Mater.* **15,** 768–774 (2016).
3. Narayan, T. C. *et al.* Direct visualization of hydrogen absorption dynamics in individual palladium nanoparticles. *Nat. Commun.* **8,** 14020 (2017).
4. Ftouni, H. *et al.* Thermal conductivity of silicon nitride membranes is not sensitive to stress. *Phys. Rev. B* **92,** 125439 (2015).
5. Baffou, G. *et al.* Photoinduced heating of nanoparticle arrays. *ACS Nano* **7,** 6478–6488 (2013).
6. Bonn, M. Phonon- Versus Electron-Mediated Desorption and Oxidation of CO on Ru(0001). *Science* **285,** 1042–1045 (1999).
7. Swearer, D. F. *et al.* Heterometallic antenna−reactor complexes for photocatalysis. *Proc. Natl. Acad. Sci.* **113,** 8916–8920 (2016).
8. Bento, A. C., Gandra, F. C. G., Da Silva, E. C., Vargas, H. & Miranda, L. C. M. Thermal diffusivity of palladium-hydrogen systems at room temperature using photothermal detection. *Phys. Rev. B* **45,** 5031–5034 (1992).
